# Prognostic value of diffuse splenic FDG uptake on PET/CT in patients with gastric cancer

**DOI:** 10.1371/journal.pone.0196110

**Published:** 2018-04-26

**Authors:** Hai-Jeon Yoon, Bom Sahn Kim, Chang Mo Moon, Jang Yoo, Ko Eun Lee, Yemi Kim

**Affiliations:** 1 Department of Nuclear Medicine, College of Medicine, Ewha Womans University, Seoul, Republic of Korea; 2 Department of Internal Medicine, College of Medicine, Ewha Womans University, Seoul, Republic of Korea; 3 Tissue Injury Defense Research Center, Ewha Womans University, Seoul, Republic of Korea; 4 Clinical Research Institute, College of Medicine, Ewha Womans University, Seoul, Republic of Korea; National Cancer Center, JAPAN

## Abstract

**Background:**

This study investigated the prognostic value of diffuse splenic uptake on F-18 fluorodeoxyglucose (FDG) positron emission tomography (PET)/computed tomography (CT) in gastric cancer (GC) patients.

**Methods:**

A total of 134 pathology confirmed GC patients who underwent PET/CT for staging work-ups were enrolled. The maximal standardized uptake value (SUV_*max*_) of primary tumor (T_*max*_), spleen (S_*max*_), and spleen to liver uptake ratio (SLR) were measured. The prognostic value of PET-measured parameters in GC patients for predicting recurrence-free survival (RFS) and overall survival (OS) were assessed. And the relationships of the parameters with hematological and inflammatory parameters were also investigated.

**Results:**

During follow-up period, 19 patients (14.1%) had disease recurrence and 12 (8.9%) died from GC. In univariate analysis, hematocrit (*p*<0.001 and *p* = 0.002), neutrophil to lymphocyte ratio (NLR; *p* = 0.021 and *p* = 0.040), AJCC staging (*p*<0.001 and *p*<0.001), adjuvant chemotherapy (*p*<0.001 and *p*<0.001), T_*max*_ (*p* = 0.004 and *p* = 0.005), and SLR (*p* = 0.005 and *p* = 0.016) were significant prognostic factors for RFS and OS, whereas platelet to lymphocyte ratio (PLR; *p* = 0.034) was a significant prognostic factor for RFS. In multivariate analysis, only SLR was an independent prognostic factor for RFS (*p* = 0.018, adjusted HR = 3.011, 95% CI = 1.207–7.511). SLR were significantly associated with serum hematocrit level (*r* = -0.256, *p* = 0.002), PLR (*r* = 0.362, *p* = 0.001), and T_*max*_ (*r* = 0.280, *p* = 0.001).

**Conclusion:**

Diffuse splenic uptake on FDG PET/CT was correlated with the level of hematological and inflammatory parameters and was an independent predictor for RFS in GC.

## Introduction

Gastric cancer (GC) is one of the most common cancers and leading cause of cancer deaths worldwide [1]. Although the survival benefit of surgical resection and adjuvant chemotherapy in GC, recurrence can occur in many cases during follow-up period. Because no effective therapy exists for recurrent setting, the estimation of the risk of recurrence has been of the clinical interest. Generally, the risk of recurrence is estimated based on various prognostic factors including TNM stage [2], histological classification [3], resection margin [4], serosal invasion [4], and tumor markers [5].

Recently, the role of the inflammation emerged as an important part of the oncogenesis [6–8]. Several studies have investigated the prognostic role of inflammatory markers, such as C-reactive protein (CRP) [9], the neutrophil to lymphocyte ratio (NLR) [10], and the platelet to lymphocyte ratio (PLR) [11] in GC.

Fluorine 18 fluorodeoxyglucose (FDG) positron emission tomography (PET)/computed tomography (CT) has been widely used for tumor evaluation, but applications for this modality are now being expanded to non-tumor pathophysiology, e.g. inflammation [12]. Based on our clinical experiences, variable range of diffuse splenic uptake compared to the liver have been identified in GC patients who were referred for staging work up. However, the clinical meaning of variable range of diffuse splenic uptake in GC patients is unexplained.

There exists one study that investigated the clinical significance of diffuse splenic uptake in heterogeneous patient group [13]. The study suggested the potential of diffuse splenic uptake as an imaging biomarker for the systemic inflammation and hematological imbalance in accordance with the significant relationship with serum inflammatory and hematological parameters. However, no studies have investigated the prognostic significance of diffuse splenic uptake in patients with GC.

In this study, we evaluated the prognostic value of diffuse splenic uptake in GC patients along with other previously reported prognostic factors. Moreover, the relationships of diffuse splenic uptake on PET/CT with serum inflammatory and hematological markers were also investigated.

## Materials and methods

### Patients and follow-up

This study was approved by the institutional review board (IRB) of the Ewha Womans University Mokdong Hospital. All procedures performed in studies involving human participants were in accordance with the ethical standards of the IRB and with the 1964 Helsinki declaration and its later amendments or comparable ethical standards. Waiver of consent was obtained from the IRB for all patients and all of the data was anonymized prior to analysis. We conducted a retrospective review of the FDG PET/CT database at Gastric Cancer Center between January 2011 and February 2016. We retrospectively enrolled 134 pathology confirmed GC patients who underwent FDG PET/CT for staging work-ups. Patients who (1) had a previous history of other malignancies; (2) received any neoadjuvant chemotherapy before PET/CT; (3) was interpreted as a false negative on PET/CT because there was no focal abnormal hypermetabolic lesion in gastric wall; (4) had a distant metastasis; (4) had acute or chronic inflammatory disease or (5) had a short follow-up time (less than 6 months) were excluded. All enrolled patients were evaluated with a physical examination, laboratory tests, and esophagogastroduodenoscopy (EGD) before surgery, and then underwent surgical resection with or without adjuvant chemotherapy. The mean time interval between preoperative PET/CT and surgery was 8.0 days (range 0.0–35.0 days).

Presence of diabetic mellitus (DM) or hypertension (HTN), social history of smoking habit or alcohol intake were checked by the review of the medical records. The results of preoperative blood tests (including blood cell counts, hemoglobin, hematocrit, and CRP) were also retrieved. The NLR and PLR were calculated using blood test results. The pathological T and N stages of the patients were evaluated according to the American Joint Committee on Cancer staging guidelines.

After surgical resection, all enrolled patients underwent routine clinical follow-up for the recurrence surveillance. Time to recurrence was defined as the time from surgery to the date of the first finding that suggested recurrence on any studies and that led to further imaging studies and/or pathologic confirmation. Time to survival was defined as the time from surgery to the date of death. Patients without recurrence or death were censored at the date of the last follow-up.

### ^18^F-FDG PET/CT and image analysis

All patients were evaluated with FDG PET/CT for staging work-ups. Before the FDG injection, patients were instructed to fast at least six hours and blood glucose level was confirmed to be<140 mg/dL. Each patient was injected with the FDG dose of 5.18 MBq/kg. After the injection of FDG, patients were strictly instructed to rest for one hour before the scan. A non-contrast CT was obtained first, and then an emission PET scan was obtained from the skull base to the thigh using an integrated Siemens Biograph mCT with 128 slice CT (Siemens Medical Solutions, Erlangen, Germany). The emission PET images were acquired for 2 min scan/bed position with 3D mode and were reconstructed with 3.0 mm slice thickness using a 3D OSEM iterative algorithm.

The PET/CT data were reviewed by 2 board-certified nuclear medicine physicians and were averaged. Both readers could refer other imaging studies including EGD, while were blinded to other clinical information. The maximal standardized uptake value (SUV_*max*_) of primary tumor (T_*max*_) was measured by placing a spheroid volume of interest (VOI) encasing the GC lesion with setting a margin threshold as 40% of the SUV_*max*_. The SUV_*max*_ of spleen (S_*max*_) was measured by placing a spheroid VOI on the center of spleen. The SUV_*max*_ of liver was measured by placing a spheroid VOI on the right lobe of the liver. Then, we calculated spleen to liver ratio (SLR) by dividing the spleen SUV_*max*_ by the liver SUV_*max*_.

### Statistical analyses

A value *p*<0.05 was regarded as statistically significant. All statistical analyses were carried out using SPSS software version 18.0.

Differences in variables between patient groups were analyzed using the Chi-square test for categorical variables or the student t-test for continuous variables. The Kaplan-Meier survival curve was generated to estimate cumulative recurrence-free survival (RFS) and overall survival (OS) rates. All continuous variables in survival analysis were dichotomized by specific cutoff values, which were resulted by the receiver operating characteristic (ROC) analysis. Survival curves were compared with the log-rank test. To evaluate the prognostic values of variables for RFS and OS, univariate and multivariate Cox proportional regression analyses were performed and hazard ratios (HR) with 95% confidence interval (CI) were obtained for variables. Prognostic variables with statistical significance in univariate analysis were included in multivariate Cox proportional regression analysis to determine independent significant factors. Before performing this analysis, some variables with high collinearity or long 95% CI were omitted. An age-adjusted Spearman partial correlation analysis was performed to identify the relationships between splenic FDG uptake with other clinical features.

## Results

A total of 134 subjects (M:F = 84:50) were included in the analysis. Table 1 summarizes the characteristics of the 134 GC patients in the study. Age (*p* = 0.022), histopathology (*p* = 0.037), AJCC staging (*p*<0.001), adjuvant chemotherapy (*p*<0.001), hematocrit (*p* = 0.014), PLR (*p*<0.001), CRP (*p* = 0.005), T_*max*_ (*p*<0.001) and SLR (*p* = 0.003) were significantly different between patients with and without recurrence, whereas the other characteristics were not different.

**Table 1:** Characteristics of patients.

Characteristic	Total (n = 134)	Recurrence (n = 19)	No Recurrence (n = 115)	*P*
Age (years)	60.6±11.8	54.9±11.7	61.5±11.7	0.022*
Male sex, n (%)	84 (62.7)	12 (63.2)	72 (62.6)	0.963
Smoking, n (%)	39 (29.1)	8 (42.1)	31 (26.9)	0.179
Alcohol intake, n (%)	48 (34.5)	10 (50.0)	38 (31.9)	0.095
DM, n (%)	27 (20.1)	4 (21.0)	23 (20.0)	0.915
Histopathology				0.037*
Differentiated	50	3	47	
Undifferentiated	84	16	68	
AJCC staging				<0.001*
I-II	96	3	93	
III	38	16	22	
Treatment				<0.001*
Surgery	95	4	91	
Surgery plus Chemotherapy	39	15	24	
Hematocrit, %	38.3±4.9	35.8±5.3	38.7±4.8	0.014*
WBC, x10^3^ cells/uL	6.8±2.3	6.4±1.9	6.8±2.3	0.444
NLR	2.4±2.3	2.9±2.7	2.3±2.2	0.346
PLR	9.3±6.6	12.0±10.1	8.8±5.8	<0.001*
CRP, mg/L	3.4±6.1	2.2±3.5	3.7±6.5	0.005*
T_*max*_	5.3±4.9	7.8±8.3	4.9±3.9	<0.001*
S_*max*_	2.4±0.3	2.4±0.4	2.4±0.3	0.085
SLR	81.2±12.4	88.8±21.9	80.0±9.5	0.003*

Differentiated, papillary or tubular adenocarcinoma. Undifferentiated, poorly differentiated or undifferentiated adenocarcinoma, or signet ring cell carcinoma

*p<0.05

### Prognostic factors for predicting RFS

**Fig 1:**
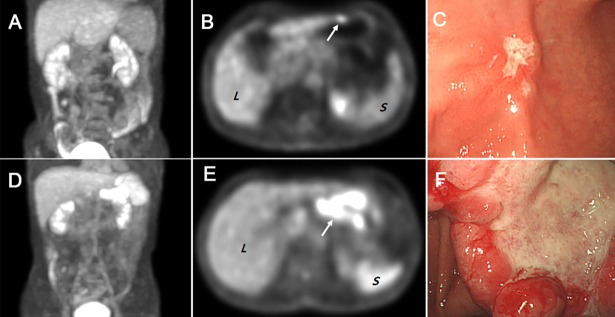
(A-C) A 53-year-old women with stomach cancer underwent FDG PET/CT. The maximum intensity projection (MIP) image (A) and transaxial PET (B) show diffuse spleen (*S*) FDG uptake lower than liver (*L*) FDG uptake. The transaxial PET also shows a focal FDG uptake at the anterior wall of stomach antrum (*arrow*), which is matched lesion with endoscopic finding (C). The calculated SLR was 55.87. After subtotal gastrectomy, final pathology confirmed stage of early gastric cancer (pT1N0M0) and histologic type of signet ring cell carcinoma. She had no recurrence during the follow up period of 48.9 months. (D-F) A 57-year-old man with stomach cancer underwent FDG PET/CT. The maximum intensity projection (MIP) image (D) and transaxial PET (E) show diffuse spleen (*S*) FDG uptake higher than liver (*L*) FDG uptake. The transaxial PET also shows a focal FDG uptake at stomach antrum (*arrow*), which is matched lesion with endoscopic finding (F). The calculated SLR was 123.31. After distal gastrectomy, final pathology confirmed stage of advanced gastric cancer (pT4aN3bM0) and histologic type of poorly differentiated tubular adenocarcinoma. He had a peritoneal recurrence after 9.1 months and finally died after 13.2 months.

**Fig 2:**
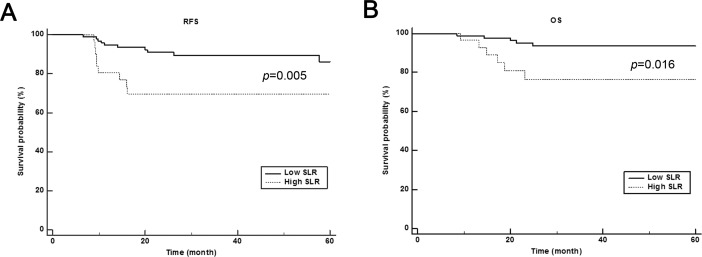
Cumulative recurrence-free survival (A) and overall survival (B) curves on the basis of spleen to liver ratio (SLR). Patients with high SLR showed significantly worse prognosis than those with low SLR values.

During the mean follow-up period of 34.5±21.8 (range, 6.5–74.8) months, 19 patients (14.1%) had disease recurrence. The optimal cutoff values for continuous variables were determined by the ROC analysis; 36.9% for hematocrit, 10.1 for PLR, 1.92 for NLR, 12.65 mg/L for CRP, 3.37 for T_*max*_, 2.5 for S_*max*_, and 86.97 for SLR. In univariate Kaplan-Meier and Cox regression analyses, hematocrit (*p*<0.001), PLR (*p* = 0.034), NLR (*p* = 0.021), AJCC staging (*p*<0.001), adjuvant chemotherapy (*p*<0.001), T_*max*_ (*p* = 0.004), and SLR (*p* = 0.005) were significant prognostic factors for the recurrence of GC. The detailed data are presented in Table 2. Before performing multivariate analysis, PLR and NLR were found to be collinear, thus only NLR was subsequently included in the multivariate model. AJCC staging, adjuvant chemotherapy, hematocrit, and T_*max*_ were found to have long 95% CI, thus the variables were not included in the multivariate model. When a multiple regression model was generated using NLR and SLR, SLR was found to be an independent prognostic factor associated with the recurrence (*p* = 0.018, HR = 3.011, 95% CI = 1.207–7.511). The representative cases of low SLR (Fig 1A–1C) and high SLR (Fig 1D–1F) with different prognosis are demonstrated. The survival curves of each SLR group are shown in Fig 2A.

**Table 2:** Predictive value of the variables for recurrence-free survival.

Variable	No. of events (%)	Mean survival (month)	Univariate *p* value	Crude hazard ratio (95% CI)	Adjusted hazard ratio (95% CI)
Age			0.326	0.635 (0.254–1.585)	
<60	11 (18.9)	70.4			
≥60	8 (10.5)	73.5			
Sex			0.959	0.856 (0.349–2.100)	
Female	7 (14.0)	70.6			
Male	12 (14.3)	73.3			
Histopathology			0.050	3.403 (0.997–11.616)	
Differentiated	3 (6.0)	79.4			
Undifferentiated	16 (19.1)	68.8			
AJCC staging			<0.001*	22.641 (6.289–81.505)^a^	
Stage I-II	3 (3.1)	81.1			
Stage III	16 (42.1)	49.9			
Adjuvant chemotherapy			<0.001*	11.721 (3.909–35.146)^a^	
(-)	4 (4.2)	80.4			
(+)	15 (38.5)	52.9			
Hematocrit			<0.001*	5.841 (2.106–16.201)^a^	
>36.9	5 (6.1)	79.8			
≤36.9	14 (26.9)	58.5			
WBC			0.094	^-^^b^	^ ^
≤9.75	19 (15.8)	71.4			
>9.75	0 (0.0)	70.8			
NLR			0.021*	3.090 (1.231–7.759)	2.540 (0.990–6.513)
≤1.92	7 (8.6)	77.1			
>1.92	12 (22.6)	64.8			
PLR			0.034*	2.890 (1.195–6.990)^c^	
≤10.1	11 (10.8)	75.4			
>10.1	8 (25.0)	57.1			
CRP			0.132	^-^^b^	^ ^
≤12.65	19 (20.9)	65.2			
>12.65	0 (0.0)	64.7			
T_*max*_			0.004*	5.227 (1.524–17.931) ^a^	
≤3.37	3 (5.4)	79.5			
>3.37	16 (20.5)	66.5			
S_*max*_			0.249	1.687 (0.685–4.156)	
≤2.5	10 (11.2)	75.1			
>2.5	9 (20.0)	67.3			
SLR			0.005*	3.379 (1.366–8.359)	3.011 (1.207–7.511)
≤86.97	10 (9.9)	76.5			
>86.97	9 (27.3)	54.8			

Univariate *p* value from log-rank test and hazard ratio from Cox proportional regression.

^a^Although significant at the univariate level, this variable has not been included in the multivariate model due to the long range of 95% CI.

^b^Hazard ratio and 95% CI cannot be calculated due to the low number of cases in each group.

^c^Although significant at the univariate level, this variable has not been included in the multivariate model because of collinearity (between PLR and NLR).

*p<0.05

### Prognostic factors for predicting OS

During the mean follow-up period of 35.0±21.5 (range, 6.5–74.8) months, 12 patients (8.9%) died from GC. In Kaplan-Meier analyses, hematocrit (*p* = 0.002), NLR (*p* = 0.040), AJCC staging (*p*<0.001), adjuvant chemotherapy (*p*<0.001), T_*max*_ (*p* = 0.005), and SLR (*p* = 0.016) were significant prognostic factors for OS. The detailed data are presented in Table 3. The survival curves of each SLR group are shown in Fig 2B.

**Table 3:** Predictive value of the variables for overall survival.

Variable	No. of events (%)	Mean survival (month)	Univariate *p* value	Crude hazard ratio (95% CI)
Age			0.225	0.482 (0.145–1.608)
<60	8 (13.8)	73.9		
≥60	4 (5.3)	77.4		
Sex			0.856	0.898 (0.292–2.764)
Female	4 (8.0)	75.2		
Male	8 (9.5)	76.5		
Histopathology			0.117	3.462 (0.765–15.664)
Differentiated	2 (4.0)	80.8		
Undifferentiated	10 (11.9)	73.9		
AJCC staging			<0.001*	52.364 (6.405–428.125)
Stage I-II	1 (1.0)	82.7		
Stage III	11 (28.9)	56.9		
Adjuvant chemotherapy			<0.001*	32.656 (4.244–251.288)
(-)	1 (1.05)	82.8		
(+)	11 (28.2)	60.3		
Hematocrit			0.002*	6.752 (1.839–24.784)
>36.9	3 (3.7)	81.7		
≤36.9	9 (17.3)	64.2		
WBC			0.178	-^a^
≤9.75	12 (10.1)	75.6		
>9.75	0 (0.0)	70.8		
NLR			0.040*	3.660 (1.126–11.899)
≤1.92	4 (4.9)	79.8		
>1.92	8 (15.1)	70.1		
PLR			0.101	3.020 (1.011–9.020)
≤10.1	7 (6.9)	78.3		
>10.1	5 (15.6)	62.8		
CRP			0.238	-^a^
≤12.65	12 (13.2)	70.3		
>12.65	0 (0.0)	64.7		
T_*max*_			0.005*	11.179 (1.450–86.183)
≤3.37	1 (1.8)	82.3		
>3.37	12 (15.4)	69.9		
S_*max*_			0.357	2.0 (0.670–5.971)
≤2.5	6 (6.7)	78.8		
>2.5	6 (13.3)	71.8		
SLR			0.016*	4.221 (1.413–12.616)
≤86.97	6 (5.9)	79.3		
>86.97	6 (18.2)	60.1		

Univariate *p* value from log-rank test and hazard ratio from Cox proportional regression.

^a^Hazard ratio and 95% CI cannot be calculated due to the low number of cases in each group.

*p<0.05

### Correlations between diffuse splenic uptake and other clinical features

**Fig 3:**
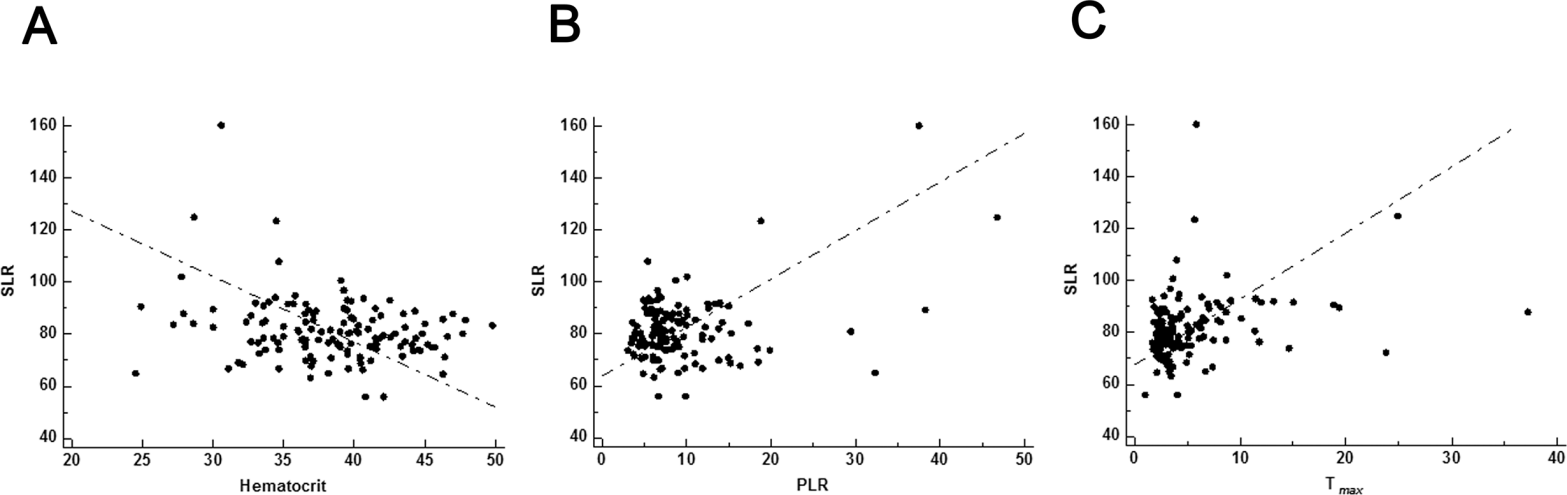
Scatter plots of serum hematocrit level (A), platelet to lymphocyte ratio (PLR) (B), and T_*max*_ (C) according spleen to liver ratio (SLR) on ^18^F-FDG PET/CT.

To identify the clinical features associated with diffuse splenic uptake, the relationships between SLR and other clinical features were assessed. After adjusting for age, SLR had a significant negative correlation with serum hematocrit level (*r* = -0.256, *p* = 0.002; Fig 3A), and a significant positive correlation with PLR (*r* = 0.362, *p* = 0.001; Fig 3B), and T_*max*_ (*r* = 0.280, *p* = 0.001; Fig 3C).

## Discussion

The present study showed that diffuse splenic FDG uptake was significantly associated with the both of RFS and OS of GC. Approximately 27.3% of patients with a high SLR experienced a recurrence, while only 9.9% of patients with a low SLR experienced a recurrence. Regarding OS, 18.2% of patients with a high SLR died from GC, while only 5.9% of patients with a low SLR died from GC. The SLR related significantly with serum hematocrit level, PLR and T_*max*_.

The clinical significance of diffuse splenic FDG uptake was studied by small number of researchers. Nam et al. investigated the relationship of diffuse splenic FDG uptake with hematological parameters and inflammatory markers based on their experience of unexplained and diffusely increased splenic FDG uptake greater than liver in some patients who performed FDG PET/CT for cancer surveillance [13]. Because the degree of splenic FDG uptake is generally less than liver, such a higher uptake compared to liver is considered as an unusual finding [14, 15]. They also used spleen/liver ratio, the corrected value of splenic uptake divided by hepatic uptake, to reduce the interindividual variation of splenic uptake. They reported that concurrent increase of serum inflammatory markers or decrease of hematologic parameters at the time of FDG PET/CT could be one of factors of diffuse splenic FDG uptake. Similarly, our results show the positive correlation between SLR and PLR, while negative correlation between SLR and serum hematocrit level.

The spleen plays important roles in mechanical filtration and creation of red blood cells, as well as active immune response to inflammation [16, 17]. The positive correlation between SLR and PLR seems to indicate an immune response by spleen to systemic inflammation in GC patients. The stimulation of megakaryocytes by proinflammatory cytokines induces thrombocytosis, thus an increased PLR has been considered as a marker of active systemic inflammation [18]. Whereas, the negative correlation between SLR and serum hematocrit level could reflect extramedullary hematopoiesis by spleen as a response to decreased serum hematocrit level.

Recently, numerous preclinical and clinical evidences have supported the link between inflammation and carcinogenesis [19]. Inflammatory response to cancer contributes to initiation, promotion, and invasion of cancer cells. The positive correlation between SLR and T_*max*_ indicates the relationship of inflammatory response with tumor aggressiveness.

Based on such a contribution of inflammation to carcinogenesis and tumor progression, the prognostic value of systemic inflammatory markers has been investigated in various type of cancers. Among the markers, NLR and PLR have been widely investigated with regards to the prognosis of GC patients [20, 21]. Increase of NLR and PLR is generally considered as a poor prognostic factor, despite the inconsistent results especially relating to the PLR [11]. In this study, we also found that high NLR and PLR were significantly related with poor prognosis on univariate analysis.

Meanwhile, anemia is a common syndrome in cancer patients and there is growing evidence to support its close relationship with poor prognosis [22, 23]. In GC, tumor bleeding, malabsorption, poor oral intake, and other factors related to tumor pathology may contribute to anemia. Similarly, our results demonstrated that low hematocrit level was significantly related with poor prognosis on univariate analysis.

On multivariate survival analysis, SLR was an independent prognostic factor for RFS, while NLR was eliminated despite the significance on univariate analysis. The results indicate the potential of diffuse splenic uptake as an integrated imaging biomarker reflecting both of systemic inflammation and hematologic imbalance.

The prognostic value of diffuse splenic FDG uptake has not been investigated in any type of cancer. A few studies just reported the prognostic significance of FDG uptake of bone marrow (BM) in patients with non-small cell lung cancer (NSCLC) [24]. The studies have reported significant relationship of BM FDG uptake with serum inflammatory markers and independent prognostic value of BLR. Because the degree of BM FDG uptake is known to move in the same direction of splenic FDG uptake on PET images, the significance of diffuse splenic uptake as prognostic factor in this study is consistent with previous studies of BM uptake.

The retrospective design and the relative small number of enrolled patients are major limitation of the current study. Some degree of selection bias could be inevitable. And, the low number of deaths in our cohort prevent the use of multivariate analyses to estimate which variables are independent predictors for OS. As shown in Table 3, crude hazard ratios of significant variables had long 95% CI due to the low number of events, and the results should be cautiously interpreted. Therefore, prospective study with a larger patient group is required to verify the prognostic role of diffuse splenic FDG uptake in cancer patients.

## Conclusions

Diffuse splenic FDG uptake would have a potential as an imaging biomarker for the systemic inflammation and hematological imbalance in GC patients considering a significant relationship with serum inflammatory and hematological parameters. The significance of diffuse splenic FDG uptake as a prognostic factor indicates a critical role of inflammation in tumor progression.
